# An evaluation of the experiences of young people in Patient and Public Involvement for palliative care research

**DOI:** 10.1177/0269216321999301

**Published:** 2021-03-17

**Authors:** Sarah J Mitchell, Anne-Marie Slowther, Jane Coad, Dena Khan, Mohini Samani, Jeremy Dale

**Affiliations:** 1 Department of Oncology and Metabolism, University of Sheffield, Sheffield, UK; 2 Warwick Medical School, University of Warwick, Coventry, UK; 3 School of Health Sciences, Queens Medical Centre Campus, University of Nottingham, UK; 4 NIHR CRN West Midlands Young Person’s Steering Group, Stafford, Birmingham, UK

**Keywords:** Palliative care, children, young people, Patient and Public Involvement, evaluation

## Abstract

**Background:**

The active involvement of patients and the public in the design and conduct of research (Patient and Public Involvement) is important to add relevance and context. There are particular considerations for involving children and young people in research in potentially sensitive and emotional subject areas such as palliative care.

**Aim:**

To evaluate the experiences of young people of Patient and Public Involvement for a paediatric palliative care research study.

**Design:**

Anonymous written feedback was collected from group members about their experiences of Patient and Public Involvement in a paediatric palliative care research study. An inductive thematic analysis of the feedback was conducted using NVivo.

**Setting / Participants:**

Young people aged 12–22 years who were members of existing advisory groups at a children’s hospital, hospice and the clinical research network in the West Midlands, UK.

**Results:**

Feedback was provided by 30 young people at three meetings, held between December 2016 and February 2017. Three themes emerged: (1) Involvement: Young people have a desire to be involved in palliative care research, and recognise the importance of the subject area.

(2) Impact: Researchers should demonstrate the impact of the involvement work on the research, by regularly providing feedback. (3) Learning: Opportunities to learn both about the topic and about research more widely were valued.

**Conclusions:**

Young people want to be involved in palliative care research, and recognise its importance. A continuous relationship with the researcher throughout the study, with clear demonstration of the impact that their input has on the research plans, are important.

**What is already known on this topic?**

- Patient and Public Involvement is important to add relevance and context to research and is a requirement for many research funders, but evaluation is inconsistent.
- Involving children and young people in research about healthcare services about their care is advocated, but little is known about their experiences.
- There are important ethical considerations when involving children and young people in research, particularly when the subject of the research is potentially sensitive, such as palliative care.

**What this paper adds**

- Young people wanted to be involved, despite the sensitive nature of the research, and were keen that their involvement had impact.
- Insights into the experiences of young people demonstrated that feedback to the group, and opportunities for learning, were valued.

**Implications for practice, theory or policy**

- There is much to learn from the experiences of Patient and Public Involvement contributors in palliative care research to inform best practice.
- The conduct of Patient and Public Involvement work, including a relationship with the researcher that allows regular feedback to contributors, is important and requires careful planning from the start of the research.
- Strategies for the evaluation of the experiences of patient and public contributors should be embedded throughout research studies and warrant further attention.

## Background

Patient and Public Involvement is defined as the active involvement of patients and members of the public in the design and process of research to ensure that it is relevant and contextual.^1,2^ The positive impact of Patient and Public Involvement in palliative care research through all stages, from the early design to translation into practice has been demonstrated.^3,4^ Challenges exist in conducting meaningful Patient and Public Involvement in palliative care research, including involving people with relevant experience and enduring perceptions amongst researchers that people may not want to be involved.^5^ Involving children and young people in research that concerns palliative care to children is important but raises specific ethical concerns.^6,7^ Currently the evidence base to inform the conduct of Patient and Public Involvement with children and young people in research is limited,^8^ with much of the published literature focussing on process^9–12^ or the experience of young people as research participants^13^ rather than their experiences. There is a range of guidance to support the conduct of Patient and Public Involvement, and tools to assess its impact, however the use of these tools, and reporting of involvement is currently variable.^14–19^

### Objective

The aim of the evaluation was to provide insights into the experiences of young people who were Patient and Public Involvement contributors to a research study in paediatric palliative care.

### Methods

This methods section outlines the Patient and Public Involvement work, personal reflections from some of the young people involved, and the method of the evaluation.

#### Description of the patient and public involvement

Young people were recruited to a Patient and Public Involvement group for a paediatric palliative care research study from three existing young people’s advisory groups in the West Midlands (based at Birmingham Children’s Hospital, the National Institute for Health Research Clinical Research Network West Midlands and Acorns Children’s Hospice). Their participation in this Patient and Public Involvement work was voluntary. They were provided with information about the research, plans for their involvement and details of opportunities including dissemination activity, through a presentation and a written leaflet. They were also provided with an information leaflet to share with their parents, which included the contact details of the researcher in case of concern. Participation in the informal evaluation reported here was also voluntary.

Arrangements to attend the group meetings were made with the group facilitator (an employed person at each organisation). The youngest contributor at the meetings was 12 years old; the oldest was 22 years old. The group included young people with interests in healthcare service design or research. Some had personal experience of palliative care services, or experiences of palliative care for a relative. The study was carried out over 5 years and contact was maintained with the group throughout, but the membership of the group evolved and changed over that timeframe as the young people’s commitments changed. One researcher (SM) conducted all of the Patient and Public Involvement work and evaluation of the experiences and perceptions of the young people involved. The work commenced in 2013 during the research application process, guiding the development of research questions from a child and family perspective. As the study progressed, contributors took part in a series of structured face-to-face sessions during which they provided advice on aspects of the study. Each session was carefully designed with the aim of gathering verbal and written feedback and advice on areas of the study including the practicalities of the study design, participant information leaflets and interview topic guides, ethical concerns, emerging new research ideas and dissemination activities.

#### Personal reflections from young people on aspects of the Patient and Public Involvement Work

Throughout the study, the young people took part in a variety of activities, providing personal reflections on their experiences as they did so. Activities included attending a research ethics committee meeting and dissemination work including co-authoring a research paper^20^ and attending conferences with posters and to give an oral presentation. The young people who took part in these activities provided written reflections, and described their experiences as being ‘*exhilarating*’ and an opportunity to feel ‘*part of something to make a difference*’, ‘*as though my voice has not just been heard, but people have listened actively*’. They valued the opportunities to ‘*share my passions around the [Young Person’s] Group*’ and ‘*learn new things*’, and particularly being ‘*regarded as highly as all the professionals [at the conference]*’. These were activities that the young people ‘*hope[d] to repeat many more times*’ and would encourage others to take part in: ‘*I would say to other young people . . . do not be scared and say how you feel and be honest. . . . if we are not asked or do not say what we feel then how are things going to change? It is being the voice of many*’.

A detailed reflection on taking part in a research ethics committee is provided in Box 1:

**Box 1.** Attending a research ethics committee, Dena Khan, PPI Co-Author.

The prospect of partaking in an ethics committee was an exciting opportunity. My understanding of clinical research has allowed me to understand the importance of ensuring any form of research is ethically sound. I want to pursue psychology and psychological research so this experience was even more valuable to me. Having no clue what to expect, I found the event insightful and interesting, although it didn’t take very long! I was able to see how important the ethics approvals process is to hold researchers to account and make sure patients/participants are remaining the central focus of any study. I was reassured that our study did not prompt a lot of ethical concerns, and I felt glad to be part of a project that takes into account both [young people’s] opinion and the welfare of those involved. Being able to go to an ethics committee has furthered my interest in research, and has made me grateful for the amount of precautions put in place. However, it has also shown me how young people can be so easily involved in research and how our opinions and ideas can be used to the benefit a study as I noticed the surprise in the committee of a young person’s presence.

#### Design of the evaluation

The evaluation was conducted during the study period at three group meetings, held between December 2016 and February 2017 (towards the end of the study, when data collection was complete, analysis was underway and dissemination plans were under consideration). This was an informal evaluation, conducted at the end of the Patient and Public Involvement sessions. The aims were firstly to gain insights into the experiences of the young people taking part in the Patient and Public Involvement work for palliative care research, and, secondly, to inform the design of future sessions. This was not research, and ethical approval was not required, however the ethical aspects of the involvement work were considered carefully and are reported elsewhere.^7^

A simple method for evaluation based on the ‘Tell Me. . .’ exercise outlined in ‘RCPCH &Us’ Recipes for Engagement^21^ was used. This involved asking group members to provide anonymous written feedback on any aspect of their Patient and Public Involvement experience related to the study by writing individual comments on post-it notes and contributing to a collection of comments from the wider group. They were asked to contribute in whatever way they felt able to, with no restriction on word count, or number of post-it notes. All comments were anonymous with no information requested that would identify an individual. This was deliberate in order to encourage honest and constructive feedback.

All of the written comments were transcribed and imported to NVivo v.11 for data management. An inductive thematic analysis was carried out, assigning every piece of feedback to a code, categorising the codes, and then grouping these categories into themes.^22^ The emerging codes were developed further through regular reflection and discussion with the research team.

## Findings

A total of 30 young people took part in the evaluation, 11 from the clinical research network, eight from the children’s hospice group and 11 from the children’s hospital group. Of these, 11 had personal experience of palliative care, including all from the children’s hospice group.

### Feedback from the group evaluation

Three overarching themes emerged from the data:

### Theme 1: Young people wish to be involved in palliative care research

Young people expressed a desire to be involved in the research despite the potentially sensitive subject area. Motivation to be involved included wanting to help others, and to make a difference:

*‘It’s amazing being involved, allows us to voice our own opinions and to be given the chance to make a difference’ (Hospice group participant)*

*‘Helps to make you feel that you are involved in helping the community’ (Hospital group participant)*

Young people recognised that they were not always included in research about sensitive subjects such as palliative care. Their comments suggested that they recognised this as an area for research and service improvement where there was variation in approach and understanding.

*‘Important as no-one wants to talk about it!’ (Clinical Research Network participant)*

*‘Really exciting! Important: so often overlooked or side-lined or delayed referral as treatment is often seen as superior to palliative care’ (Clinical Research Network participant)*

One young person particularly valued the opportunity to contribute to palliative care research as a way to have her opinions heard:

*‘Being asked about palliative care is very interesting because as a young person I am interested in what happens to my [relative] and the choices that are made and I am not normally involved when I would like to be. This research project gets my own opinions and thoughts about palliative care which is good as it means I am involved and listened to for once.’ (Hospice group participant)*

### Theme 2: Young people want to see that their contributions have impact on the research

The young people expressed an expectation that researchers would listen to them and provide feedback on how their advice had influenced the research. The researcher’s attendance at a series of meetings, rather than just one, was identified as important.

*‘Ensure we receive feedback and follow through throughout the project’ (Clinical Research Network participant)*

Young people valued the ongoing relationship between the researcher and the group, with the development of rapport, an open approach allowing the discussion of sensitive topics, and the opportunity to feedback on how the research was progressing.

*‘[The Patient and Public Involvement work] has been conducted in a way that makes me comfortable to contribute’ (Hospice group participant)*

### Theme 3: Young people described learning from their experiences of Patient and Public Involvement

Young people described the opportunity to learn through the Patient and Public Involvement process as a benefit. There was feedback to suggest that young people valued the opportunity to learn not only about the topic, but also about different research skills including dissemination:

*‘I think this project is very interesting and I can’t wait to hear more about this. I don’t know much about palliative care so I’m keen to learn more about it’. (Hospital group participant)*

*‘It’s good to do a research project because it gives you knowledge of the subject and you know you’re helping someone or something’ (Clinical Research Network participant)*

## Discussion

### Main findings

This evaluation provides insights into the experiences of young people who provided Patient and Public Involvement to a paediatric palliative care research study. The evaluation suggested that young people valued the opportunity to be involved, wanted their involvement to have impact, and learnt from their involvement.

### Strengths and limitations

There is no widely accepted method for the evaluation of the experiences of Patient and Public Involvement contributors.^23^ This evaluation took a pragmatic approach, adopting a method of engagement previously devised and tested by young people that was quick and simple to conduct and appeared acceptable to the groups. While this approach allowed detailed anonymous feedback to be collected, a more rigorous and systematic approach could be used to evaluate the experiences of Patient and Public Involvement in future research studies.

The Patient and Public Involvement work described in this report was conducted prior to the 2020 COVID-19 pandemic. While many of the activities described could be adapted for online sessions, careful consideration should be given to methods of evaluation. Similar work in the future is likely to require the use of online questionnaires or interactive presentation tools that would allow researchers to engage an online audience in real time. Young people are likely to be able to suggest solutions. Furthermore, the ability to conduct sessions online may provide opportunities to involve a wider and more diverse group of young people than those who were included in this evaluation who were all already members of organisational advisory groups.

### What this study adds

There are very few studies describing the involvement of young people in palliative care research. This evaluation provides new insights into the perspectives of young people involved in a palliative care research study. The findings of the evaluation support published guidance highlighting the importance of involving young people in research about their care.^17,24^ The insights provided should encourage researchers to involve young people, despite the potentially sensitive nature of palliative care research. Previous research suggests that young people do not want their involvement to be tokenistic,^25,26^ and researchers can be criticised if they fail to engage or update young people as the research progresses.^27^ These challenges can be overcome through the development of relationship between the researcher and the group over the time course of the study, with regular feedback and updates on the progress of the study and the impact of their involvement.

## Conclusion

There is an ongoing need to share examples of best practice Patient and Public Involvement in research, to ensure that approaches are robust and meaningful. This pragmatic evaluation suggests that young people value and benefit from the opportunity to learn new skills and about new subjects in palliative care research. The success of Patient and Public Involvement can depend on a continuous relationship with the researcher, allowing time for feedback, and for young people to understand how they are making a difference.
